# Vitamins Modulate the Expression of Antioxidant Genes in Progesterone-Treated Pancreatic *β* Cells: Perspectives for Gestational Diabetes Management

**DOI:** 10.1155/2020/8745120

**Published:** 2020-09-15

**Authors:** Nathália Ruder Borçari, Jeniffer Farias dos Santos, Gustavo Roncoli Reigado, Bruna Letícia Freitas, Mariana da Silva Araújo, Viviane Abreu Nunes

**Affiliations:** ^1^Department of Biotechnology, University of Sao Paulo (USP), Sao Paulo, Brazil; ^2^Department of Biochemistry, Federal University of Sao Paulo (UNIFESP), Sao Paulo, Brazil

## Abstract

Gestational diabetes (GD) is a condition defined as carbohydrate intolerance and hyperglycemia beginning in the second trimester of pregnancy, which overlaps with the progesterone exponential increase. Progesterone has been shown to cause pancreatic *β*-cell death by a mechanism dependent on the generation of reactive oxygen species and oxidative stress. Herein, we studied the effect of this hormone on the expression of 84 genes related to oxidative stress and oxidant defense in pancreatic RINm5F cell lineage. Cells were incubated with 0.1, 1.0, or 100 *μ*M progesterone for 6 or 24 h, in the presence or absence of the vitamins E and C. Among the investigated genes, five of them had their expression increased, at least 2-fold, in two different concentrations independently of the time of incubation, or at the same concentration at the different time points, including those that encode for stearoyl-CoA desaturase 1 (*Scd1*), dual oxidase 1 (*Duox1*), glutathione peroxidase 6 (*GPx6*), heme oxygenase 1 (*Hmox1*), and heat shock protein a1a (*Hspa1a*). Vitamins E and C were able to increase, in progesterone-treated cells, the expression of genes with antioxidant function such as *Hmox1*, but decreased *Scd1* expression, a gene with prooxidant function. At cytoplasmic level, progesterone positively modulated *Hmox1* and *Hspa1a* content. These results suggest that the protein encoded by these genes might protect cells against progesterone induced-oxidative damage, opening perspectives to elucidate the molecular mechanism involved in progesterone action in GD, as well as for the development of antioxidant strategies for the prevention and treatment of this disease.

## 1. Introduction

During pregnancy, carbohydrate metabolism is highly affected, including increased insulin synthesis and secretion, as well as enhanced peripheral resistance to this hormone, *β*-cell proliferation, and increasing in pancreatic islet volume [1, 2], an essential aspect for glycaemia regulation during pregnancy. However, the inability of maternal islets to respond to the increased insulin demand may lead to the development of gestational diabetes (GD).

GD is a condition characterized by carbohydrate intolerance, resulting in hyperglycemia, associated with insulin resistance and decreased pancreatic *β*-cell function [3]. It is correlated to maternal and perinatal morbidity, increased type 2 diabetes mellitus, cardiovascular disease, and metabolic syndrome risks, being a public health problem [4, 5]. Several evidences suggest that insulin resistance and inflammation, as well as obesity, might play an important role in the onset of this condition [6].

The pregnancy comprises a drastic increase in the concentration of progesterone, a hormone that has been associated to the increased peripheral insulin resistance [7, 8] and in the GD development [7, 9]. Additionally, Picard et al. [10] and Nadal et al. [11] suggested that the hormonal peculiarities of pregnancy, specifically the high progesterone concentrations in the second trimester of pregnancy, which overlaps with the GD onset, may contribute to insufficient adaptation in the insulin secretion during this period [12–14].

Regarding progesterone effects on different cell types, Verma and Rana [15] found high lipid peroxidation in the liver and kidneys of benzene-treated rats after receiving doses of this hormone. Cheng et al. [16] have demonstrated that progesterone was capable of contributing to TNF-*α*-mediated apoptosis by a mechanism involving the generation of free radicals in HuH-7 hepatoma cells. Additionally, Ito et al. [17] showed, using mitochondria isolated from the liver of Wistar rats, that progesterone participates in the oxidative stress through the formation of superoxide anion (O_2_^•^) and hydrogen peroxide (H_2_O_2_).

We have already shown that progesterone induces apoptotic death of RINm5F *β* pancreatic insulin-producing cells and rat islets [18]. We also found that progesterone-induced cell death was significantly reduced when cells were preincubated with vitamin E, corroborating the hypothesis that the effect of this hormone involves a molecular mechanism associated with the oxidative stress.

Considering this complex scenario, we hypothesized that progesterone could regulate the expression of genes involved in oxidative stress and antioxidant defense in pancreatic *β* cells, which could be related to its mechanism of action triggering survival or death pathways in these cells. Here, we have shown that progesterone upregulated the expression of the genes that encodes for the stearoyl-CoA desaturase 1 (*Scd1*), dual oxidase 1 (*Duox1*), glutathione peroxidase 6 (*GPx6*), heme oxygenase 1 (*Hmox1*), and heat shock protein a1a (*Hspa1a*).

Although little is known about *Scd1* involvement in GD pathophysiology, Azevedo-Martins and Miyazaki [19] reported that protein encoded by this gene participates in metabolic control and its inhibition could be favorable for diabetes treatment, obesity, and other metabolic diseases [20]. Also, previous studies have shown that *Scd1*-deficient mice are protected from insulin resistance, hypertriglyceridemia, hepatic steatosis, and diet-induced and genetically induced obesity [21].

*Duox1* belongs to NADPH oxidase (NOX) family enzyme, well known to be a source O_2_^•^ and H_2_O_2_. The relationship between *Duox1* and GD is poorly explored; however, it has been shown that the increased NOX-induced reactive oxygen species (ROS) production is associated to diabetic retinopathy, and the vitamins E and C have been shown to reduce the vascular injury in this condition [22].

*GPx6* is a gene that encodes to a ROS detoxification enzyme. However, there are not specific data regarding this gene in GD context, and it has been shown that the concentration of the enzyme encoded by the *GPx3* gene remained high between the 16^th^ and 20^th^ and between the 32^nd^ and 36^th^ weeks in pregnant women with GD compared to the healthy ones, possibly to compensate the excessive ROS generation [23]. Additionally, Zygula et al. [24] showed that the *GPx* and glutathione transferase activities were higher in the plasma of insulin-treated GD pregnant in comparison to healthy pregnant women.

Accordingly, an important relationship between progesterone and *Hmox1* has been suggested by Zenclussen et al. [25]. Also, Yang et al. [6] investigated *Hmox1* involvement in GD scenario and demonstrated that low *Hmox1* serum concentrations at the beginning of pregnancy are related to higher risk for GD development.

*Hspa1a* is a gene that belongs to the heat shock protein (HSP) family, which members exhibit antioxidant properties and play an important anti-inflammatory role [26]. The association between *Hspa1a* and GD is still poorly understood; however, Garamvölgyi et al. [27] demonstrated that serum *Hspa1a* concentrations were significantly higher in pre-GD and GD than in healthy pregnant women. Furthermore, studies have demonstrated that circulating HSP70 concentrations are elevated in patients with type 1 or 2 diabetes mellitus [28–31].

The presented data might contribute to a better understanding of the pathogenesis of GD, opening new perspectives not only to elucidate the molecular mechanism involved in the progesterone action on pancreatic cells and its relationship with GD, but also to the development of therapeutic strategies for this disease based on antioxidant approaches.

## 2. Material and Methods

### 2.1. Cell Cultures

RINm5F insulin-producing cells, a cell lineage derived from rat insulinoma (ATCC, American Type Culture Collection, CRL-11605), and MDA-MB-231 cells (ATCC® HTB-26™), a human breast cancer cell line, were maintained in RPMI-1640 supplemented with 24 mM sodium bicarbonate, 2 mM glutamine, and 20 mM HEPES. The human breast cancer cell line MCF7 (ATCC® HTB-22™) was maintained in DMEM containing 4 mM glucose. Both media were supplemented with 10% fetal bovine serum and the antibiotics 10 U/ml penicillin and 10 mg/ml streptomycin. Cells were cultivated under a humidified atmosphere at 37°C and 5% CO_2_. MCF7 and MDA-MB-231 were used as positive and negative controls for progesterone receptor expression, respectively, in the cell death experiments.

### 2.2. Cell Treatments

RINm5F (2 × 10^5^), MDA-MB-231 (2 × 10^5^), and MCF7 (2 × 10^5^) cells were seeded into 6-well plates 48 h before the incubation with progesterone in different concentrations (0.1, 1.0, or 100 *μ*M, diluted in absolute ethanol) or with only absolute ethanol (control) in a final volume of 2 ml, by 6 or 24 h in the culture conditions. The effect of the vitamins E and C on progesterone-treated RINm5F cells was studied on the cell death and on the expression of *Hmox1*, *Prdx4*, and *Scd1* genes, based on evidences of their modulation by exogenous antioxidants [32–34]. Also, we investigated the role of these vitamins on the ROS generation in progesterone-treated cells. In the experiments with antioxidants, cells were preincubated for 2 h with (or without) 40 *μ*M vitamin E (dissolved in absolute ethanol) or 50 *μ*M vitamin C (diluted in water) prior progesterone addition, and after the indicated periods of time, cultures were subjected to the different experiments. Progesterone concentrations were chosen considering different scenarios: 0.1 *μ*M corresponds to the physiological concentration of the hormone in a nonpregnant condition and at the beginning of pregnancy; 1.0 *μ*M refers to a physiological concentration that can be reached in the second and third trimesters of pregnancy; and 100 *μ*M corresponds to a pharmacological concentration of the hormone used to prevent preterm delivery.

### 2.3. Cell Viability and DNA Fragmentation Analysis

After incubation with progesterone, in the presence or absence of antioxidants vitamin E or C, cells were collected by trypsinization (0,25%, Invitrogen, CA, USA), centrifuged at 400 × *g* for 7 min at 4°C and the pellet was suspended in 300 *μ*l phosphate-buffered saline (PBS). For cell viability analysis, 5 *μ*l of propidium iodide (PI) solution (1 mg/ml in PBS, Invitrogen, CA, USA) was added to the cells. For DNA fragmentation analysis, pellets were suspended in 300 *μ*l PBS containing 0.1% Triton X-100 (Sigma-Aldrich Corporation, St. Louis, USA) and 20 *μ*g/ml PI. Cells were analyzed by a Guava flow cytometer (Millipore Corporation, Hayward, CA, USA) using the InCyte software (Millipore Corporation, Hayward, CA, USA). A total of 10,000 events were acquired.

### 2.4. RNA Extraction, cDNA Synthesis, and Quantitative PCR

RNA was extracted using the Mini Kit RNeasy (Qiagen, Frederick, MD, USA). The complementary DNA (cDNA) was obtained by reverse transcription, using 8 *μ*l of RNA, 4 *μ*l of the 5X reaction buffer (BC3), 1 *μ*l of oligo (dT)_18_, 2 *μ*l of transcriptase reverse enzyme (RE3), and 3 *μ*l of RNase free water, in 20 *μ*l of final volume. This mixture was incubated for 15 min at 42°C and, then for 5 min at 95°C in a thermocycler, according to the RT^2^ First Strand Kit (Qiagen, Hilden, German). The effect of progesterone on the expression of 84 genes involved in oxidative stress and antioxidant defense was analyzed by quantitative PCR, using the RT^2^ Profiler™ PCR Array Human Oxidative Stress (PAHS-065Z) (Qiagen, Hilden, German) according to the manufacturer's instructions. The cycle in which the reaction crosses the detection threshold (cycle threshold–Ct) of the interest genes (IG) was correlated to amount of the target mRNA. Standardization was performed by the expression of constitutive genes (CG) provided by the kit (HPRT (hypoxanthine-guanine phosphoribosyl transferase), *β*-2 microglobulin, and *β*-actin). The Ct value of CG was subtracted of the Ct value of IG, resulting in ΔCt value, which represents the relative amount of the IG transcripts. The increment was calculated as 2^−ΔΔCt,^ considering the gene expression in the control samples (cells cultured with absolute ethanol and the vehicle used for progesterone preparation). Data related to the gene expression were presented in terms of the fold increasing in comparison to the gene expression in control. Following PCR Array experiments, gene expression was better studied by quantitative PCR by using the primers: *Hmox1*F : GACAGCATGTCCCAGGATTT,*Hmox1*R : ATGGTACAAGGAGGCCATCA;*Prdx4*F : CTTTTGGGGATCGAATTGAA, *Prdx4*R : AATCCTTATTGGCCCCAGTC;*Scd1*F TCAATCTCGGGAGAACATCCT,*Scd1*R : TCGATGAAGGTGGTGAA.PrimerF sequences for *Scd1*, *Hspa1a*, and *Prdx4* amplification were designed by Qiagen (Hilden, German) and acquired commercially. Reactions were performed using the SYBR® Green PCR Master Mix Kit (Thermo Fisher Scientific, Waltham, MA, USA) in 40 thermal cycles at Eco Real-Time PCR System (Illumina, San Diego, CA, USA) using 1 *μ*l cDNA (125 ng/*μ*l), 4 *μ*l of different sets of primers (10 *μ*M), and 5 *μ*l of Master Mix.

### 2.5. Criteria for Gene Selection for Studies

To allow better evaluation of the genes potentially involved in the progesterone action on pancreatic *β* cells, different selection steps were performed, based on criteria specifically defined for this study. The first selection step considered genes whose expression was modulated minimum twice by progesterone (positively or negatively) in, at least, two different concentrations, regardless of the time of incubation with the hormone (6 or 24 h), or at the same progesterone concentrations at both time points. Then, from 29 selected genes, five were chosen because they are related or have functions related to pancreatic *β* cells and/or DG.

### 2.6. Immunodetection of *Hmox1* and *Hspa1a* Proteins by ELISA

After cell treatment, total proteins were obtained by adding 300 *µ*l of lysis buffer (PBS, 0.1% Triton X-100 and a cocktail of protease and phosphatase inhibitors P-8340, Sigma-Aldrich, USA). The amount of protein in each sample was determined using a standard bovine serum albumin (BSA) curve. *Hmox1* concentration was determined using the Human Total HO-1/HMOX1 DuoSet IC ELISA (enzyme-linked immunosorbent assay) kit DYC3776-2 (R&D Systems Inc., Minneapolis, USA) according to the manufacturer instructions. Initially, a 96-well microplate was covered with the capture *Hmox1* antibody (8.0 *μ*g/ml) diluted in PBS, sealed and incubated overnight at room temperature. After this period of incubation, the antibody solution was removed and the wells were washed three times with 300 *μ*l of wash buffer. Nonspecific binding sites were blocked with 300 *μ*l of blocking buffer, followed by the incubation for 2 h at room temperature. After blocking, the wells were washed again three times with 300 *μ*l of wash buffer, and 100 *μ*l of standard *Hmox1* solution (supplied by the kit, 156 to 10,000 pg/ml) was added for the standard curve determination. In a second step, 100 *μ*l of progesterone-treated cell extracts was used instead of the standard *Hmox1* solution. After three washes with 300 *μ*l of wash buffer, 100 *μ*l of the *Hmox1* detection antibody (200 ng/ml) solution was added. The plate was sealed and incubated for 2 h at room temperature. After plate wash, 100 *μ*l of horseradish peroxidase streptavidin-conjugated antibodies was added and 20 min incubation at room temperature was performed. Thereafter, the wells were washed with 300 *μ*l of wash buffer followed by the addition of 100 *μ*l of the substrate solution, which was incubated for 20 min in a light protected chamber. After this incubation, 50 *μ*l of the stop solution was added. The absorbance of the samples was determined at 450 nm in a Synergy HT plate reader (Biotek, Winooski, VT, USA) using Gen5™ software (Biotek, Winooski, VT, USA). *Hspa1a* concentration was determined using Human Total HSP70/HSPA1A DuoSet IC ELISA (enzyme-linked immunosorbent assay) kit DYC1663-2 (R&D Systems Inc., Minneapolis, USA) according to the manufacturer's instructions and following the same procedure described above for *Hmox1* immunodetection. *Scd1*, *Duox1*, *GPx6*, and *Prdx4* protein content was also investigated, but they were not easily detectable by the available antibodies.

### 2.7. Intracellular ROS Production Measurement

ROS generation was indirectly determined by DCFDA fluorescence-based assay [35, 36]. The cells were seeded in a 96-well culture plate (density of 2 × 10^4^ cells/well), and after 1 day of culture, they were pretreated for 2 h with 40 *μ*M vitamin E (dissolved in absolute ethanol) or 50 *μ*M vitamin C (diluted in water) prior to 0.1, 1.0, or 100 *μ*M progesterone incubation for 3, 6, or 24 h. For intracellular ROS indirectly quantification, the cells were incubated with the DCFDA dye (10 *μ*M in PBS) for 20 min, at 37°C in dark conditions, which is deacetylated by cellular esterases to a nonfluorescent compound and later oxidized by ROS into 2′, 7′–dichlorofluorescein (DCF). DCF fluorescence was continuously measured at *λ*_excitation_ = 480 nm and *λ*_emission=_  = 530 nm in a Synergy microplate reader (Biotek Instruments, EUA) for 60 min. The slopes of curves were converted into mean velocity of DCF formation and expressed as mean ± standard error (SEM) in arbitrary units of fluorescence (AUF)/min. After, the results were normalized to the number of viable cells. Experiments were performed, at least, three times in quadruplicates.

### 2.8. Statistical Analysis

Statistical analysis was performed by one- and two-way ANOVA plus Bonferroni post-tests for multiple comparisons using GraphPad Prism 5.01. Unless specified, results were expressed as means ± SEM of, at least, three individual experiments in duplicates and compared to control (cells cultivated with absolute ethanol) or to the cultures incubated in the absence of vitamins. Differences were considered significant at *ρ* < 0,05.

## 3. Results

### 3.1. Progesterone and Antioxidants Effect of RINm5f Cell Viability and Death

**Figure 1:**
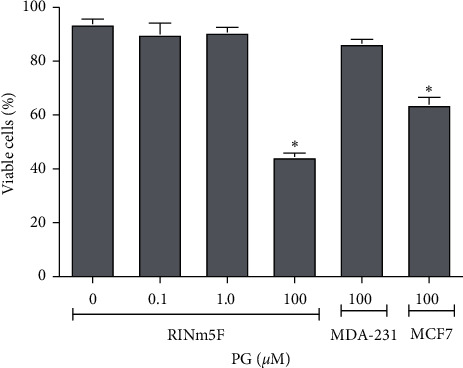
Cell viability upon progesterone treatment. RINm5F cells were analyzed by flow cytometry after incubation with 0.1, 1.0, or 100 *μ*M progesterone or absolute ethanol (control) by 24 h, and 10,000 events were analyzed per sample. Data are presented as means ± SEM of three experiments in duplicates. *∗* indicates difference in comparison to control. PG: progesterone; MDA-MB-231: negative control; MCF7: positive control for progesterone receptors expression.

**Figure 2:**
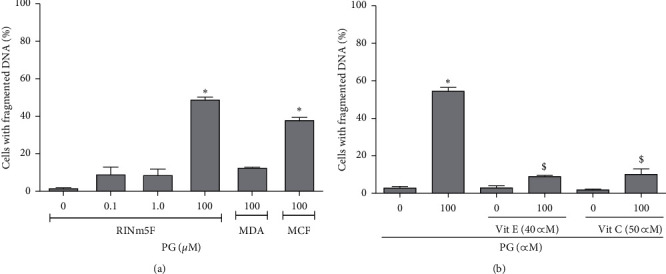
DNA fragmentation in progesterone-treated cells. (a) RINm5F cells incubated with absolute ethanol (control) or 0.1, 1.0, and 100 *μ*M progesterone by 24 h. (b) Cells were preincubated with 40 *μ*M vitamin E or 50 *μ*M vitamin C for 2 h and then with absolute ethanol (control) or 100 *μ*M progesterone for 24 h. 10,000 events were analyzed, by flow cytometry, per sample. Data are presented as means ± SEM of three experiments in duplicates. *∗* indicates difference in comparison to control. $ indicates difference between preincubation with the vitamins and the treatment with only 100 *μ*M progesterone (*ρ* < 0.05). PG: progesterone; Vit E: vitamin E; Vit C: vitamin C.

In the incubation of RINm5F cells with progesterone in different concentrations for 24 h, there was significant loss of membrane integrity in 50% of cells incubated with 100 *μ*M progesterone (Figure 1), indicating that this hormone is able to cause cell necrosis in this condition, but it was not toxic at the physiologic concentrations of 0.1 and 1.0 *μ*M. Similar results were obtained with MCF7 cells (positive control), but not with MDA-MB-231 lineage, which do not express progesterone receptors (negative control).Also, when the hormone was used at the concentration of 100 *μ*M, it caused DNA fragmentation, an indicative of apoptosis occurrence, in approximately 50% of RINm5F (Figure 2) and MCF7 cells (positive control), but not in MDA-MB-231 lineage. The percentage of cells with fragmented DNA was reduced by 78 and 71% when the RINm5F cells were preincubated with 40 *μ*M vitamin E or 50 *μ*M vitamin C, respectively, prior to progesterone treatment, in comparison to control without vitamins. This result suggested a protective effect of these antioxidants against progesterone-induced cell apoptosis (Figure 2(b)).

### 3.2. Selection of Progesterone-Modulated Genes

The gene expression was determined by the calculation of fold increasing in the expression in comparison to control, considering the average expression of the constitutive genes. From 84 evaluated genes, 29 were selected based on the changes in their expression in, at least, two different concentrations in any period of time (6 or 24 h), or at the same concentrations in both time points (Table 1). Thereafter, five genes associated with pancreatic *β*-cell function and/or GD were selected (Table 2). *Scd1* and *Duox1* genes encode proteins with prooxidant properties, while *GPx6*, *Hmox1*, *Hspa1a*, and *Prdx4* genes encode proteins with antioxidant properties, as indicated. Although *Prdx4* was not regulated more than twice by progesterone, this gene was included in our analysis since it has been studied in the diabetes context, more specifically in the insulin synthesis regulation also improving endoplasmic reticulum folding capacity under high insulin requirement conditions [37, 38].

From 84 investigated genes, 29 were chosen based on the changes in their expression in at least two different progesterone concentrations in any period of time (6 or 24 h) or at the same progesterone concentrations in both time points, in comparison to control (relative expression). Negative values indicate gene down regulation. Bold letters indicate genes that were selected for further studies.

Six selected genes were grouped according to redox potential expressed as prooxidant (P) or antioxidant (A). ^*∗*^The *Prdx4* gene was not modulated by progesterone according to defined criteria, but it was included since it has been studied in the diabetes context and *β*-cell physiology. The numbers correspond to relative expression in comparison to control. Negative values refer to downregulated genes.

### 3.3. Progesterone Regulates Gene Expression in Pancreatic *β* Cells

**Figure 3:**
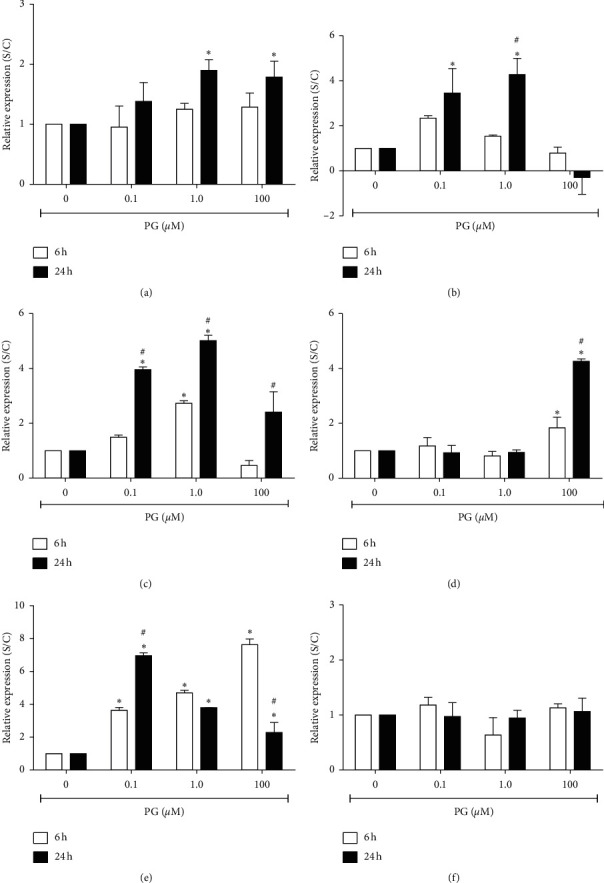
Relative expression of progesterone-regulated genes in RINm5F cells. Cells were incubated, for 6 or 24 h, with 0.1, 1.0, or 100 *μ*M progesterone. Data are presented as means ± SEM of three experiments in duplicates. (a) *Scd1*; (b) *Duox1*; (c) *GPx6*; (d) *Hmox1*; (e) *Hspa1a*; (f) *Prdx4*. S/C: expression in the progesterone-treated cultures in comparison to control. ^*∗*^ indicates difference compared to control. ^#^ indicates difference between the time points, for the same progesterone concentration (*ρ* < 0.05). PG: progesterone.

*Scd1* expression was positively modified by approximately 2-fold in comparison to the control in the incubation with 1.0 and 100 *μ*M progesterone for 24 h (Figure 3(a)), suggesting that the effect of the hormone on expression of *Scd1* was mostly time dependent.

Expression of *Duox1* was also increased about 4-fold in incubation with 0.1 or 1.0 *μ*M progesterone for 24 h. However, progesterone at the final concentration of 100 *μ*M promoted a considerable decrease in this gene expression. Furthermore, the hormone did not alter *Duox1* expression in the incubation for 6 h (Figure 3(b)).

The gene encoding for the *GPx6* protein had its expression increased up to 5-fold after incubation with progesterone for 24 h, and a milder effect was observed on the incubation of cells with the hormone for 6 h (Figure 3(c)).

The gene encoding for *Hmox1* had its expression increased by approximately 2-fold after incubation of cells with 100 *μ*M progesterone by 6 h and about four times in the incubation for 24 h with the hormone at this same concentration, commonly used to prevent preterm delivery (Figure 3(d)).

Specifically, *Hspa1a* was the best modulated gene by progesterone, and its expression was modified approximately four and seven times in incubation with 0.1 *μ*M progesterone, by 6 and 24 h, respectively. In the incubation of cells with the hormone at a final concentration of 1.0 *μ*M, by 6 or 24 h, it was verified an increase on the expression of this gene of five and four times, respectively, and in the incubation with 100 *μ*M progesterone, by 6 and 24 h, there was an increase of about eight and three times, respectively, in comparison to control (Figure 3(e)).

### 3.4. Expression of *Scd1*, *Hmox1*, and *Prdx4* Genes in the Presence of Antioxidants

**Figure 4:**
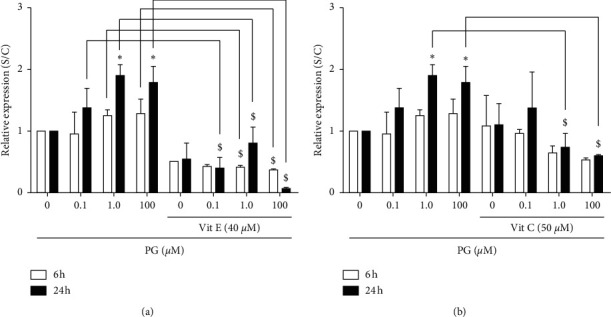
Expression of *Scd1* gene in progesterone-treated RINm5F cells, in the presence of antioxidants. Cells were preincubated with 40 *μ*M vitamin E (a) or 50 *μ*M vitamin C (b) for 2 h, and then with 0.1, 1.0, or 100 *μ*M progesterone 6 or 24 h. Data are presented as means ± SEM of three experiments in duplicates. ^*∗*^ indicates difference compared to control. ^#^ indicates difference between the incubation time points, for the same progesterone concentration. $ indicates difference in comparison to the treatment without antioxidants (*ρ* < 0.05). PG: progesterone; Vit E: vitamin E; Vit C: vitamin C.

As it was already shown, *Scd1*, which is a gene encoding for a prooxidant protein, had its expression significantly increased in incubation of cells with 1.0 and 100 *μ*M progesterone by 24 h. This expression was significantly reduced, in terms of RNA, in the presence of vitamin E (Figure 4(a)). Specifically, vitamin E caused a reduction from 50 to 90% in the *Scd1* expression in progesterone-treated cells. In cells exposed to 100 *μ*M progesterone, this vitamin almost completely abolished *Scd1* expression. Following vitamin C preincubation, *Scd1* expression was decreased by approximately 50% in the treatment with 1.0 or 100 *μ*M progesterone by 24 h (Figure 4(b)).

**Figure 5:**
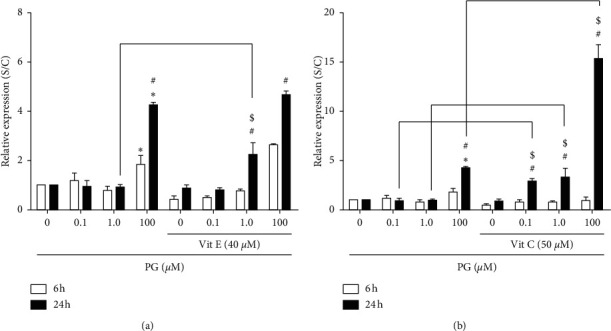
Expression of *Hmox1* in progesterone-treated RINm5F cells, in the presence of antioxidants. After preincubation with 40 *μ*M vitamin E (a) or 50 *μ*M vitamin C (b) for 2 h, cells were incubated, for 6 or 24 h, with 0.1, 1.0, or 100 *μ*M progesterone. Data are presented as means ± SEM of three experiments in duplicates. ^*∗*^ indicates difference compared to control. ^#^ indicates difference between the incubation time points, for the same progesterone concentration. $ indicates difference in comparison to the treatment without antioxidants (*ρ* < 0.05). PG: progesterone; Vit E: vitamin E; Vit C: vitamin C.

In Figure 5(a), it can be observed that vitamin E promoted a 2-fold increase in the expression of *Hmox1*, a gene with antioxidant properties, in the incubation of cells with 1.0 *μ*M progesterone, in comparison to the same treatment without this vitamin. Similarly, vitamin C promoted a significant increase in *Hmox1* expression in the incubation of cells with 0.1, 1.0, and 100 *μ*M progesterone for 24 h by approximately 3-fold (Figure 5(b).

**Figure 6:**
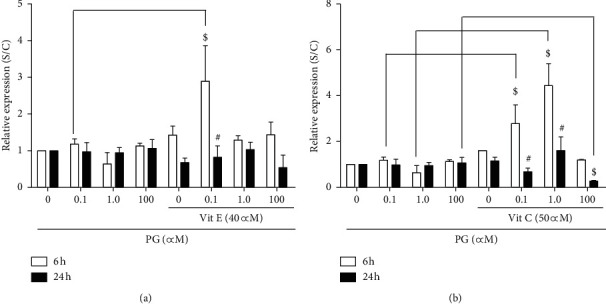
Expression of *Prdx4* in progesterone-treated RINm5F cells, in the presence of antioxidants. After preincubation with 40 *μ*M vitamin E (a) or 50 *μ*M vitamin C (b) for 2 h, cells were incubated, for 6 or 24 h, with 0.1, 1.0, or 100 *μ*M progesterone. Data are presented as means ± SEM of three experiments in duplicates. ^*∗*^ indicates difference compared to control. ^#^ indicates difference between the incubation time points, for the same progesterone concentration. $ indicates difference in comparison to the treatment without antioxidants (*ρ* < 0.05). PG: progesterone; Vit E: vitamin E; Vit C: vitamin C.

Regarding *Prdx4* expression, a gene that encodes for a protein with antioxidant functions, it was verified that vitamin E promoted an increase of almost 3-fold in the expression of this gene in the incubation with 0.1 *μ*M progesterone for 6 h (Figure 6(a)), while vitamin C caused an increase of approximately 4-fold in cells incubated with 1.0 *μ*M progesterone for 6 h (Figure 6(b)). On the other hand, this vitamin caused a decrease in the *Prdx4* expression in the incubation of cells with 100 *μ*M progesterone for 24 h, in comparison to the treatment with only the hormone.

### 3.5. Progesterone Increased *Hmox1* and *Hspa1a* at Cytoplasmic Level

**Figure 7:**
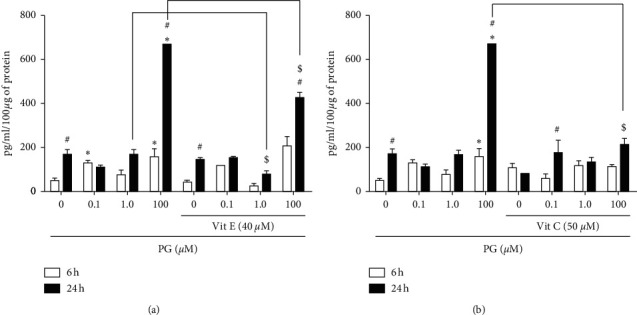
*Hmox1* content in progesterone-treated RINm5F cells. Cells were preincubated with 40 *μ*M vitamin E (a) or 50 *μ*M vitamin C (b) for 2 h, and then with 0.1, 1.0, or 100 *μ*M progesterone for 6 or 24 h. Data are presented as means ± SEM of three experiments in duplicates. ^*∗*^ indicates difference compared to control. ^#^ indicates difference between the incubation time points, for the same progesterone concentration. $ indicates difference in comparison to the treatment without antioxidants (*ρ* < 0.05). PG: progesterone; Vit E: vitamin E; Vit C: vitamin C.

To understand if the gene expression could be correlated with the protein amount, the cytoplasmic content of *Hmox1* and *Hspa1a* was determined by ELISA. Cells incubated with 0.1 and 100 *μ*M progesterone for 6 h showed an increase in *Hmox1* concentration of approximately two and three times, respectively. In the incubation with 100 *μ*M progesterone for 24 h, it was verified a significant increase of approximately four times in comparison to control (Figure 7(a)). The treatment with vitamin E promoted a decrease in the amount of *Hmox1* protein in approximately 50% when cells were incubated with 1.0 and 100 *μ*M progesterone for 24 h (Figure 7(a)). Similarly, in the presence of vitamin C, it was observed a reduction of approximately three times the amount of *Hmox1* in the incubation with 100 *μ*M progesterone for 24 h, in comparison to the treatment with only the hormone (Figure 7(b)).

Regarding *Hspa1a* protein cytosolic content, incubation of cells with 0.1, 1.0, or 100 *μ*M progesterone, for 6 h, resulted in a significant increase of 3-, 6-, and 15-fold in *Hspa1a* concentration in comparison to control, respectively, similar to the gene expression data. In the incubation with progesterone for 24 h, in all tested concentrations, there was also a significant increase in protein amount in comparison to control (Figu3re 8), which was higher than the incubation by 6 h, indicating a possibly accumulation of this protein during the time course of progesterone exposure.

### 3.6. Intracellular ROS Production Determination

**Figure 9:**
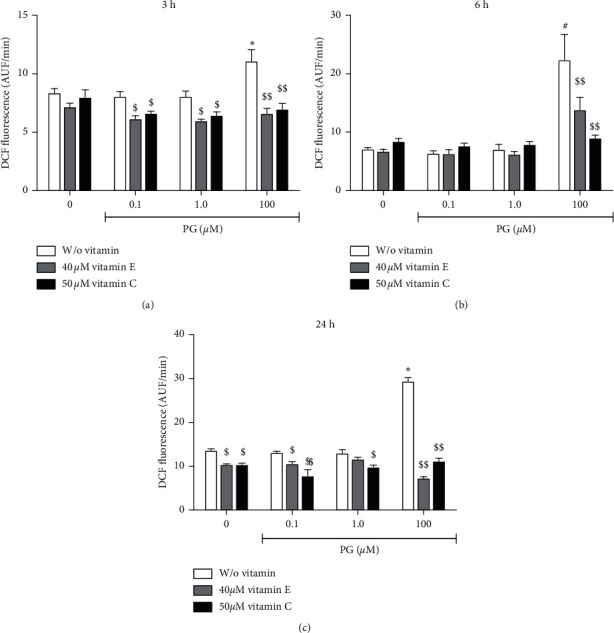
ROS content measurement in progesterone-treated RINm5F cells. Cells were preincubated with 40 *μ*M vitamin E or 50 *μ*M vitamin C for 2 h, and then with 0.1, 1.0, or 100 *μ*M progesterone for 3 (a), 6 (b), or 24 h (c). ROS content was measured by DCFDA probe (10 *μ*M) during 60 min. Data are expressed as mean ± SEM of DCF formation (AUF/min) of three experiments in quadruplicates, after normalization to the number of viable cells in each sample. ^*∗*^ indicates difference in comparison to control without progesterone. $ and $$ indicate difference in comparison to the treatment without antioxidants (*ρ* < 0.05 and *ρ* < 0.001, respectively). PG: progesterone; Vit E: vitamin E; Vit C: vitamin C.

In order to verify if the vitamins E and C were able to reduce progesterone-induced ROS generation and oxidative stress in this simplified cell culture model, which could be related to the regulation of investigated genes, we indirectly determined the ROS production using the DCFDA probe (Figure 9). Data showed that incubation of cells with 100 *μ*M progesterone for 3 h significantly increased by 30% the ROS amount in comparison to control cultures (cells incubated with absolute ethanol). Incubation of cells with 0.1 and 1.0 *μ*M progesterone did not affect ROS generation at this time point; however, the vitamins E and C significantly diminished ROS in about 20% in the cells exposed to these physiological progesterone concentrations. The ROS amount was better reduced by the vitamin E (40%) and vitamin C (37%) in the treatment of cells with 100 *μ*M progesterone (*p* < 0.001) (Figure 9(a)). Following the incubation for 6 h, it can be observed, based on the fluorescence signal, that the ROS formation was 2-fold higher than the previous time point in the incubation with 100 *μ*M progesterone, but it was not verified to the incubations with 0.1 and 1.0 *μ*M progesterone. Both vitamins E and C caused an important reduction by 38 and 60%, respectively, in the ROS formation in the 100 *μ*M progesterone-treated cultures (Figure 9(b)). Regarding to the incubation period of 24 h, control cultures or cells incubated with 0.1 and 1.0 *μ*M progesterone appeared to be basally more stressed than those treated for only 3 or 6 h, even in the presence of vitamins E (up to 90%) and C (up to 50%) (Figure 9(c)). However, vitamin E promoted a significant reduction of ROS by 20% in the incubation with 0.1 *μ*M progesterone in comparison to control without vitamin, and vitamin C diminished ROS formation by 40 and 25% in the incubation with 0.1 and 1.0 *μ*M progesterone, respectively. Incubation of cells with 100 *μ*M progesterone resulted in 3.5-fold enhancement in the ROS production in comparison to the incubation period of 3 h. Both vitamins E and C reduced ROS generation by 75 and 60%, respectively, compared to the cultures incubated with progesterone at the same concentration but without the antioxidants.

## 4. Discussion

The mechanisms by which progesterone exerts its effects on pancreatic *β* cells are still unclear; however, oxidative stress appears to be involved in the toxicity of this hormone [15, 18, 39]. Based on these evidences, we investigated the effect of progesterone on the expression of genes involved in the oxidative stress and antioxidant defense in pancreatic *β* cells, in the presence or absence of antioxidant vitamins. We observed that progesterone was able to modulate the expression of several genes, including those that encode to *Scd1, Duox1, GPx6, Hmox1*, and *Hspa1a* proteins in RINm5F insulin-producing cells.

In our investigation, it was observed an increase of approximately 2-fold in the *Scd1* expression in the incubation with progesterone for 24 h, which could represent a potential pathway for progesterone-induced oxidative stress on pancreatic *β* cells. Similarly, Marks et al. [40] observed that *Scd1* protein levels were increased in rats treated with estradiol and progesterone, being demonstrated that ROS, including hydroxyl, peroxyl, and other molecules, could act as regulators of the hormone actions and signaling pathways. We also observed that in cells preincubated with vitamin E, there was a reduction in *Scd1* expression for all tested progesterone concentrations, in both time points (6 or 24 h). Interestingly, this vitamin, in the incubation of cells with 100 *μ*M progesterone, virtually suppressed *Scd1* expression, suggesting that vitamin E could be able to attenuate progesterone-induced oxidative stress in pancreatic *β* cells. Vitamin C showed similar behavior to vitamin E on the *Scd1* expression, indicating a potential effect of these vitamins in the redox balance reestablishment in the cells. Particularly, we have shown that both vitamins E and C were able to protect pancreatic *β* cells against oxidative-stress-induced apoptotic death mediated by progesterone, which could be related to the attenuation of prooxidant gene expression and, consequently, ROS production, protecting insulin synthesis and secretion, as well as pancreatic cell proliferation [18].

Still regarding the regulation of prooxidant genes, we showed that progesterone, at the physiological concentrations, was able to increase *Duox1* expression, although its expression in RINm5F cells was relatively low. At the higher progesterone concentration (100 *μ*M), there was no increase in the expression of *Duox1* suggesting that in this condition, which has been demonstrated to be cytotoxic for RINm5F cells, DUOX does not contribute directly to ROS production. Instead, ROS can be generated by alternative mechanisms that are potentially involved in the oxidative stress pathways triggered by pharmacological progesterone doses on pancreatic *β* cells.

Among the progesterone-regulated antioxidant genes, *GPx6* had its expression enhanced up to 5-fold by the hormone. This result suggests that *GPx6* may participate in the redox balance in the cells exposed to progesterone in both studied physiological scenarios, or when this hormone is administered to prevent preterm delivery. In this direction, despite *GPx6* has not been shown to be regulated by steroid hormones, the *GPx3* expression by progesterone has been demonstrated *in vivo* from 2 to 12 h after the hormone treatment [41]. Specifically, progesterone induced *GPx3* expression through PR/HIF1*α* in mouse endometrial stromal cells, where the high level of *GPx3* was closely associated with the hydrogen peroxide reduction.

The expression of the gene encoding for *Hmox1* was also increased 4-fold by 100 *μ*M progesterone. The increase in *Hmox1* expression could represent a protective mechanism against oxidative stress important to the reestablishment of the redox status in pancreatic cells. Similarly, Zhao et al. [42] have shown that *Hmox1* expression was regulated in pancreatic islets under stress conditions and the *Hmox1* protein was involved in the production of three compounds with cytoprotective effects.

In the presence of vitamin E, *Hmox1* had its expression increased by approximately 2-fold in the incubation with 1.0 *μ*M progesterone. Also, vitamin C promoted an increase in the expression of this gene up to 15 times in the incubation with 100 *μ*M progesterone for 24 h, suggesting that these vitamins could provide additional protection to pancreatic cells against oxidative damage by increasing *Hmox1* expression. Accordingly, a study by Reed et al. [33] showed that vitamin E supplementation protected proximal renal tubules against ROS effects by increasing *Hmox1* expression.

At cytoplasmic level, 100 *μ*M progesterone was shown to promote an increase in *Hmox1* content in the incubation for 6 or 24 h, confirming the results observed at mRNA level. In the presence of the vitamins E or C, however, it was not observed the increase in the amount of *Hmox1* protein, which may suggest that the period for protein synthesis regulation by these vitamins may be higher than for gene expression modulation, which will be further investigated in future studies by the extension of the incubation time.

In the following, we studied *Hspa1a* expression and showed its positive regulation in terms of gene and cytosolic protein expression in the incubation with progesterone for 6 h, in a concentration dependent manner; in the incubation with progesterone for 24 h, there was a significant decrease of *Hspa1a* expression, but *Hspa1a* protein amount seems to reflect its accumulation in cytosolic compartment in relatively high concentrations in comparison to control or the incubation period of 6 h. These results indicate that progesterone-induced oxidative stress is able to enhance *Hspa1a* expression, which might attenuate oxidative damage, in spite of cell population having not been not completely rescued from death.

Comparably, Damsteegt et al. [43] showed an increase in *Hspa1a* expression following endoplasmic reticulum stress in MIN6 cells, a mouse pancreatic *β* cell line, after exposure to palmitate, a free saturated fatty acid well known to exert deleterious effects leading *β* cell to dysfunction and apoptosis. Using RINm5F cells, Bellmann et al. [44] showed that the treatment with *Hspa1a* protein or the transfection with *Hspa1a* gene resulted in the reduction of cell lysis induced by NO• and ROS. Corroborating these results, Burkart et al. [45] observed that the suppression of *Hspa1a* expression abolished protection against NO• effect.

*Prdx4*, which belongs to the peroxiredoxin family (*Prdx*) [46, 47], was investigated in this study based on evidences that the elimination of its expression normally renders cells more susceptible to oxidative stress-induced cell death, whereas their overexpression protects them [37, 38]. Interestingly, *Prdx4* plasma levels were shown to be significantly higher in individuals with type 2 diabetes mellitus than in nondiabetic patients [48], which could represent a mechanism to compensate oxidative stress in this condition. However, this gene was shown to be not very well expressed and modulated by progesterone in the PCR array experiments. Similar to our results, it has been demonstrated that PRDX2, −4, and −5 proteins are expressed in small amounts in cells and they are not easily detectable by antibodies. In addition, the low expression of this gene could be related to the high susceptibility of RINm5F cells to oxidative stress [49].

Nevertheless, our data showed that, in the presence of vitamin E, cells treated with 0.1 *μ*M progesterone for 6 h displayed an increase in *Prdx4* expression of approximately 3-fold compared to the treatment without the antioxidant, which may constitute a protective mechanism against ROS. Vitamin C promoted a similar effect on *Prdx4* expression in cells treated with the physiological progesterone concentrations for 6 h. Although few studies have evaluated the effect of antioxidants on *Prdx4* expression, it was already shown that proteins from this family, such as *PRDX2*, −3, and −6, had their expression negatively regulated when human lymphocytes were exposed to H_2_O_2_, whereas this effect was reversed after treatment with vitamin E [50].

Finally, we investigated if the vitamins E and C were able to reduce progesterone-induced ROS generation and oxidative stress in RINm5F cells, which could be related to direct action of these antioxidants on subcellular structures and redox processes, in addition to the regulation of the investigated genes. The data corroborated the findings of our group in which we have shown that progesterone at a final concentration of 100 *μ*M caused a significant enhancement of ROS production in these cells [18]. Here, we have shown that the vitamins E and C significantly diminished ROS in RINm5F cells being this effect best observed in the cell cultures exposed to 100 *μ*M progesterone, a condition that causes death in more than 50% of cell population and triggers the upregulation of prooxidant genes such as *Scd1* and *Duox1*. Specifically, we showed that the vitamins E and C significantly reduced the expression of *Scd1*, suggesting that this effect could also result in decreased rates of ROS formation.

In summary, we showed that progesterone can regulate the expression of several genes related to oxidative stress and antioxidant defense in the pancreatic *β* cells, which may be related, directly or indirectly, to the GD establishment or development. Particularly, the increase in *Hmox1* gene expression might be associated with the cell protection against oxidative damage caused by progesterone, being considered a therapeutic target for diabetes and other oxidative stress diseases [34]. In addition, the increased expression of genes encoding for proteins with prooxidant characteristics such as *Scd1* e *Duox1* could be related to the mechanism of action of this hormone. Interestingly, expression of *Scd1* was virtually abolished by the presence of vitamin E in progesterone-treated cells. We also showed that the vitamins E and C, easily obtained through the diet, were able to increase the expression of antioxidant genes and decrease the expression of prooxidant genes, which may constitute an efficient mechanism of cellular defense against oxidative damage.

Together, our results allow us to access several novel aspects about pancreatic *β*-cell physiology, in addition to contribute to a better understanding of the molecular mechanism involved in the action of progesterone on these cells, opening perspectives for the development of strategies of prevention and treatment of GD, based on the reestablishment of the redox state of the maternal organism and the use of the antioxidant therapy.

## Figures and Tables

**Figure 8:**
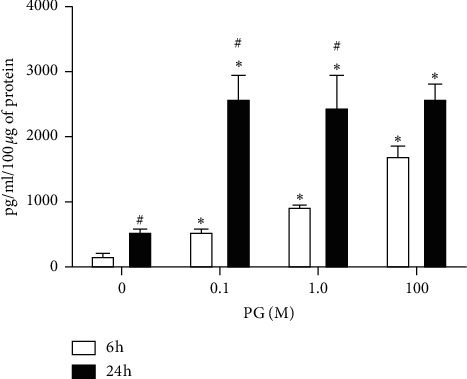
*Hspa1a* content in progesterone-treated RINm5F cells. Cells were incubated with 0.1, 1.0, or 100 *μ*M progesterone for 6 or 24 h. Data are presented as means ± SEM of three experiments in duplicates. ^*∗*^ indicates difference in comparison to control. ^#^ indicates difference between 6 and 24 h (*ρ* < 0.05). PG: progesterone.

**Table 1:** Regulated genes in progesterone-treated RINm5F cells evaluated by PCR array.

Gene	6 h			24 h			
	Progesterone concentrations						
	0.1 *μ*M	1 *μ*M	100 *μ*M		0.1 *μ*M	1 *μ*M	100 *μ*M
*Cyba*	2.17	1.50	−1.46		2.69	2.33	1.27
*Dhcr24*	−1.55	−1.45	−5.77		−1.36	−2.13	−1.72
*Dnm2*	−1.51	−1.56	−5.46		−1.48	−2.04	−2.10
**Duox1**	**2.45**	**1.59**	**1.45**		**4.54**	**2.84**	**-1.04**
*Ercc6*	−1.11	−1.38	−3.61		−1.20	−1.48	−2.14
*Fancc*	−1.27	−1.53	−3.31		−1.34	−2.04	−2.86
*Fmo2*	1.15	−2.01	−1.76		5.55	5.89	1.96
*Gpx2*	−1.13	−1.17	−2.39		1.15	−1.11	−2.33
**Gpx6**	**1.57**	**2.82**	−**1.15**		**4.06**	**4.82**	**1.12**
*Gpx7*	1.03	−1.83	−2.35		3.07	2.53	2.87
**Hmox1**	−**1.09**	−**1.57**	**2.66**		−**1.26**	−**1.63**	**4.18**
**Hspa1a**	**3.81**	**4.85**	**7.98**		**7.14**	**3.80**	**1.24**
*Idh1*	1.05	−1.03	−2.56		1.28	1.34	−2.20
*Lpo*	−2.81	1.20	−1.33		1.90	3.62	23.08
*Ncf1*	−1.03	−1.16	−3.81		−1.19	−1.33	−3.68
*Ncf2*	−1.60	1.04	−14.08		1.53	−4.72	1.95
*Ngb*	−1.51	−1.56	−4.64		1.76	2.29	−1.28
*Noxa1*	−2.37	−1.22	−7.60		−1.26	−1.90	−2.15
*Noxo1*	1.17	−1.71	−7.83		−1.36	1.09	−2.47
*Nqo1*	−1.28	−1.70	−9.26		−1.22	−1.82	3.18
*Prnp*	−1.56	−2.23	−2.10		−1.46	−2.01	−2.70
*Ptgs1*	−1.33	−1.71	−2.15		−1.55	−2.05	−1.96
*Idh1*	3.73	4.60	2.09		1.10	1.35	3.08
**Idh1**	−**1.11**	**1.39**	−**1.42**		**2.00**	**2.15**	**1.38**
*Slc38a1*	2.20	4.75	−1.66		1.92	2.96	−2.90
*Srxn1*	−1.11	−1.13	−3.61		−1.09	−1.23	4.63
*Txnip*	−1.16	−1.56	−50.94		−2.19	−3.53	−26.65
*Ucp2*	−1.62	−1.79	−4.51		−1.40	−1.73	−2.10
*Vim*	1.58	1.01	−7.99		1.29	2.55	−1.14

**Table 2:** Genes modulated by progesterone in RINm5F cells.

Gene	Pot. redox	6 h			24 h			
		Progesterone concentrations						
		0.1 *μ*M	1 *μ*M	100 *μ*M		0.1 *μ*M	1 *μ*M	100 *μ*M
*Scd1*	P	−1.11	1.39	−1.42		2.00	2.15	1.38
*Duox1*	P	2.45	1.59	1.45		4.54	2.84	−1.04
*Gpx6*	A	1.57	2.82	−1.15		4.06	4.82	1.12
*Hmox1*	A	−1.09	−1.57	2.66		−1.26	−1.63	4.18
*Hspa1a*	A	3.81	4.85	7.98		7.14	3.80	1.24
*Prdx4* ^*∗*^	A	1.25	1.22	1.07		1.66	1.42	1.06

## Data Availability

Previously reported data were used to support this study and are available at DOI: 10.1530/JOE-13-0202. This prior study of our group is cited at relevant places within the text as the reference Nunes et al. [18].
